# Farmers' Perceptions About Health and Welfare Issues in Turkey Production

**DOI:** 10.3389/fvets.2020.00332

**Published:** 2020-06-12

**Authors:** Nienke van Staaveren, Emily M. Leishman, Benjamin J. Wood, Alexandra Harlander-Matauschek, Christine F. Baes

**Affiliations:** ^1^Centre for the Genetic Improvement of Livestock, Department of Animal Biosciences, University of Guelph, Guelph, ON, Canada; ^2^The Campbell Centre for the Study of Animal Welfare, Department of Animal Biosciences, University of Guelph, Guelph, ON, Canada; ^3^Hybrid Turkeys, Kitchener, ON, Canada; ^4^School of Veterinary Science, University of Queensland, Gatton, QLD, Australia; ^5^Institute of Genetics, Vetsuisse Faculty, University of Bern, Bern, Switzerland

**Keywords:** turkey, farmer, attitude, mortality, culling, condemnation, health, welfare

## Abstract

Farmers play an essential role in the management of animals and ensuring their health and welfare. However, relatively little is known about the health and welfare-related issues farmers themselves find important in the turkey sector. As part of a larger study, a cross-sectional survey of turkey farmers was conducted in Canada to identify the main perceived reasons for culling, mortality, and carcass condemnations in their flocks. Additionally, farmers were asked to rate the importance of different health and welfare-related issues (i.e., mortality, aggressive pecking, disease, leg injuries, leg deformities, breast injuries, and varying body size) during their summer and winter production, as well as for the sector as a whole. A total of 83 responses were analyzed (response rate 20%). The most frequently mentioned reasons for the culling of turkeys included leg-related issues (90.0%), sickness (60.5%), and small body size (58.0%). The perceived reasons for mortality were most often unknown (59.7%), or related to cannibalism (41.6%) or dehydration (42.9%). The main reasons for carcass condemnations at processing were related to skin (33.8%) or subcutaneous conditions (64.7%). Leg deformities and mortality were considered the biggest issues for the turkey production sector. In general, farmers rated items as more of an issue when the question pertained to the sector as a whole rather than to their farm. These results increase our understanding of the health and welfare-related problems in turkey production that farmers find important. This can ultimately help focus research efforts in addressing these issues through improved management adaptations or breeding approaches, thereby improving both the well-being of farmers and birds.

## Introduction

Farm animal health and welfare is an important component of agriculture. The United Nations recently recommended improvements in animal health and welfare as a way toward sustainable agriculture, recognizing the connections between animal welfare and sustainability, economic development, food security, human nutrition, human health, and human wellbeing (1–4). As such, farm animal welfare is important to different stakeholders ranging from farmers and farmer organizations to policy-makers, veterinarians, breeding companies, and the public (4–7). However, farmers, as the primary care-takers of animals, play a crucial role in managing animal health and welfare (8, 9) and can be a valuable source of knowledge (10).

There are several animal health and welfare-related issues that can play a role within turkey farming, including but not limited to disease susceptibility and management, injurious pecking and aggression, footpad dermatitis, and leg abnormalities (11). Those issues not only represent health and welfare problems within the flock but can also lead to financial losses due to decreased productivity, carcass condemnations, and downgrading of carcass value at processing (12). Additionally, such problems can negatively impact how consumers view turkey farming (13). This can possibly strain the relationship between farmers and consumers, who hold farmers responsible for ensuring animal health and welfare (14, 15). Consequently, health and welfare issues on turkey farms can have implications for farmer wellbeing, livelihood, and job satisfaction. And, for those reasons, individual farmers can benefit from improvements in turkey health and welfare (3, 4).

Previous work has mainly focused on identifying risk factors in the social and physical environment of the birds in efforts to improve turkey health and welfare (12, 16–19). However, there is limited information available on what turkey farmers themselves consider important for turkey health and welfare (20). Specifically, what kind of turkey health or welfare-related issues they struggle with, or how they perceive these issues is unknown. This information can increase our understanding of the issues and possible barriers that need to be overcome to improve turkey health and welfare. Farmers who do not perceive certain animal health or welfare problems as an issue are less likely to undertake actions to try and correct these problems. For example, farmers' willingness to reduce aggressive behavior was influenced by their perception of aggression as a problem in pigs (21), and farmers differ in what threshold they consider acceptable for levels of injurious pecking in laying hens (22). Furthermore, farmer attitudes and behavior can impact animal fear, stress, and productivity (23, 24) as shown, for instance, by Kielland et al. (25) and Cransberg et al. (26). They found that farmers with a more positive attitude toward animals had a lower prevalence of skin lesions in dairy herds and less fearful broilers with higher productivity (25, 26). No such findings have thus far been reported in turkey farming. Considering that farmers are responsible for decisions and changes in the day-to-day management that influence animal health and welfare (8, 9), it is crucial to understand how health and welfare-related issues are perceived by turkey farmers.

## Materials and Methods

Turkey farmers were asked to take part in a cross-sectional survey as part of a larger project to identify Canadian housing and management factors (van Staaveren et al., in preparation) and associate these practices with pecking injuries and footpad dermatitis in turkeys (Leishman et al., in preparation). The survey covered sections on general farm information, housing aspects, litter management, feed and water management, flock health, and farmer perceptions. The current study describes results arising from the section of the survey focusing on farmers' perceptions of turkey health and welfare-related issues either on their farm or the turkey production sector as a whole. Specifically, farmers were asked to indicate the main perceived reasons for culling (pecking injuries, sick, small body size, leg injuries [e.g., lameness broken legs], leg deformities [e.g., varus, valgus], pendulous crop, breast buttons/blisters, wing injuries, other) and mortality (dehydration, smothering, cannibalism, disease, mechanical failure, unknown, other) in their current flock. Culling was defined as the process of removing birds from the farm based on specific criteria, while mortality refers to the death of a bird on-farm (27). Farmers also provided the reasons for carcass condemnations or downgrading (respiratory conditions, sub-cutaneous conditions, leg injuries, leg deformities, skin conditions, fluid in the abdomen, liver conditions, emaciation, dark-colored carcasses, ruptured tendons, other) at processing based on slaughterhouse records from previous flocks. Farmers could select multiple reasons for culling, mortality and carcass condemnations.

Furthermore, farmers were asked to rate how they perceived health and welfare-related issues such as mortality, aggressive pecking, disease, leg injuries, leg deformities, breast injuries, and varying body size, on their farm in summer and winter. Ratings ranged from “no issue,” “a small issue,” or “a big issue.” Finally, they were asked to provide a similar rating for these issues for the sector as a whole. In the questionnaire, terminology was adapted to improve farmers' comprehension where possible. For instance, examples of leg deformities were described by Canadian farmer-specific terms as “hockey stick legs” (valgus: feet turned outward, and hocks turned inward) and “cowboy legs” (varus: feet turned inward, and hocks turned outward) (28). Surveys were made available in hard-copy and online (Qualtrics, Provo, UT, USA) format and were distributed to all registered turkey farmers (~500 farmers) across Canada through the Turkey Farmers of Canada to ensure data were collected anonymously. Invitations started in April 2019, and the Turkey Farmers of Canada sent out regular reminders until the end of data collection in December 2019. This study was approved by the University of Guelph Research Ethics Board (REB19-02-015).

Data were entered into Microsoft Office Excel using manual double entry and checked for entry errors. Invalid responses were considered as missing values. Frequency of responses was calculated using descriptive statistics in SAS v9.3 (SAS Inst. Inc., Cary, NC). Multinomial models were developed for each health and welfare condition to determine the likelihood of farmers giving a higher issue rating (higher rating indicating the condition being a larger issue) in summer compared to winter, or on their farm compared to the sector as a whole (SAS Inst. Inc., Cary, NC). Results are presented as odds ratio (OR) and their 95% confidence interval (95%CI), with an OR > 1 indicating higher odds for a higher rating for a condition which reflects that it is considered as more of an issue.

## Results

A total of 83 questionnaires were returned (20% response rate). More of the farmers answered the survey for hen flocks (64%) compared to a smaller proportion of farmers who represented tom flocks (36%). It should be noted that not all farmers answered all questions, and therefore the final sample size per question can differ.

### Perceived Reasons for Culling, Mortality and Carcass Condemnation

**Figure 1:**
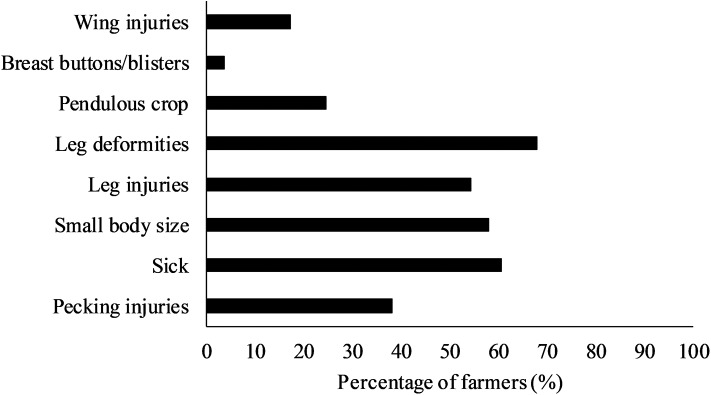
Percentage (%) of farmers (*n* = 81) that selected different reasons for culling in their turkey flock. Farmers could select multiple options.

**Figure 2:**
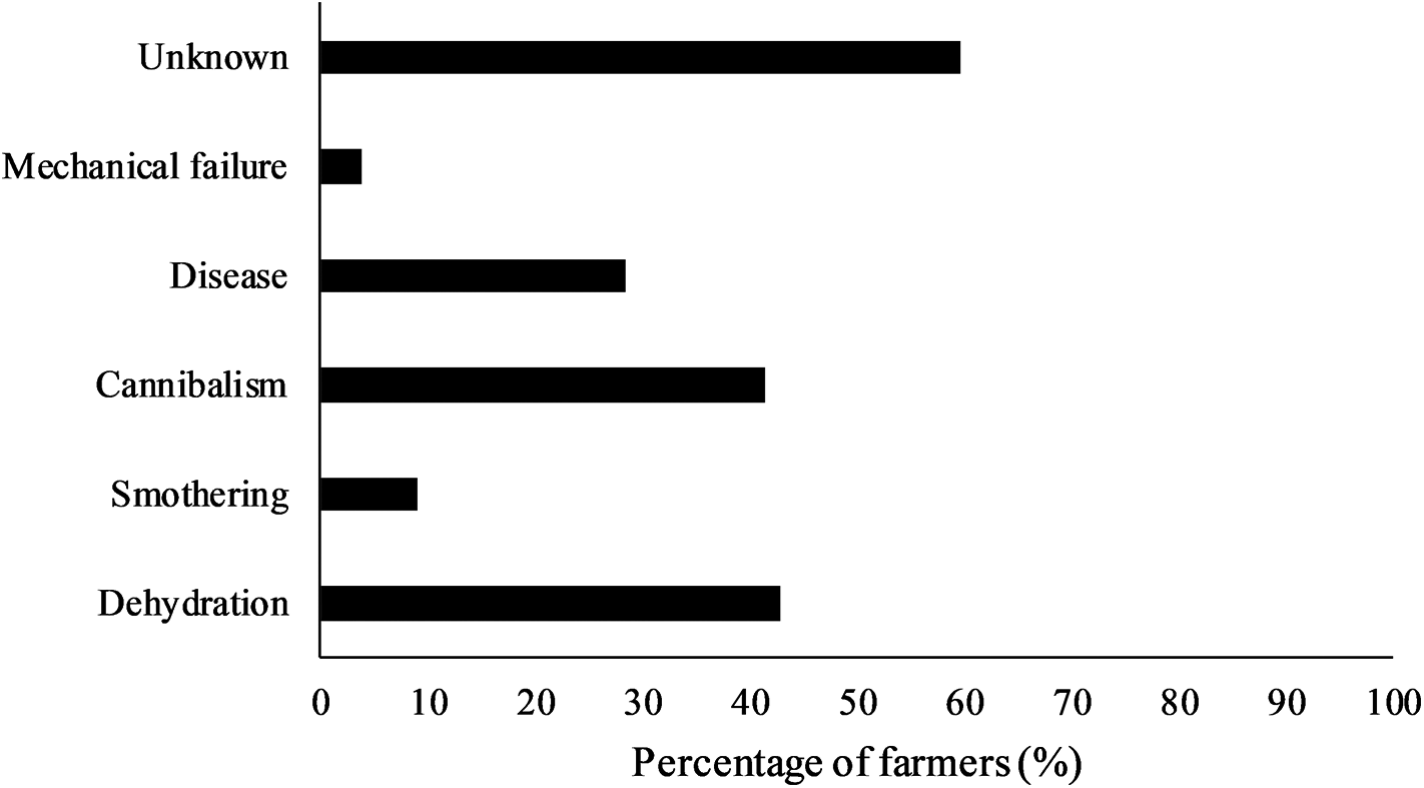
Percentage (%) of farmers (*n* = 77) that selected different causes of mortality in their turkey flock. Farmers could select multiple options.

Farmers were asked to indicate the main perceived reasons for culling and mortality in their flocks, as shown in Figures 1, 2, respectively. The most frequently selected reason for culling of turkeys involved leg deformities (67.9%), followed by leg injuries by 54.3% of the farmers. Additionally, birds that showed signs of being sick (60.5%; unrelated to skeletal issues) and birds with small body size relative to their age (58%) were frequently mentioned as reasons for culling. More than a third of farmers also indicated pecking injuries (38.3%) as a reason for culling, while pendulous crop (24.7%), wing injuries (17.3%), and breast buttons/blisters (3.7%) were less frequently mentioned (Figure 1).

In contrast, the perceived causes of mortality were unknown in the majority of cases (59.7%). The other main causes of mortality included dehydration (42.9%), cannibalism (41.6%), and disease (28.6%). Smothering (9.1%) and mechanical failure (3.9%) were mentioned infrequently (Figure 2).

**Figure 3:**
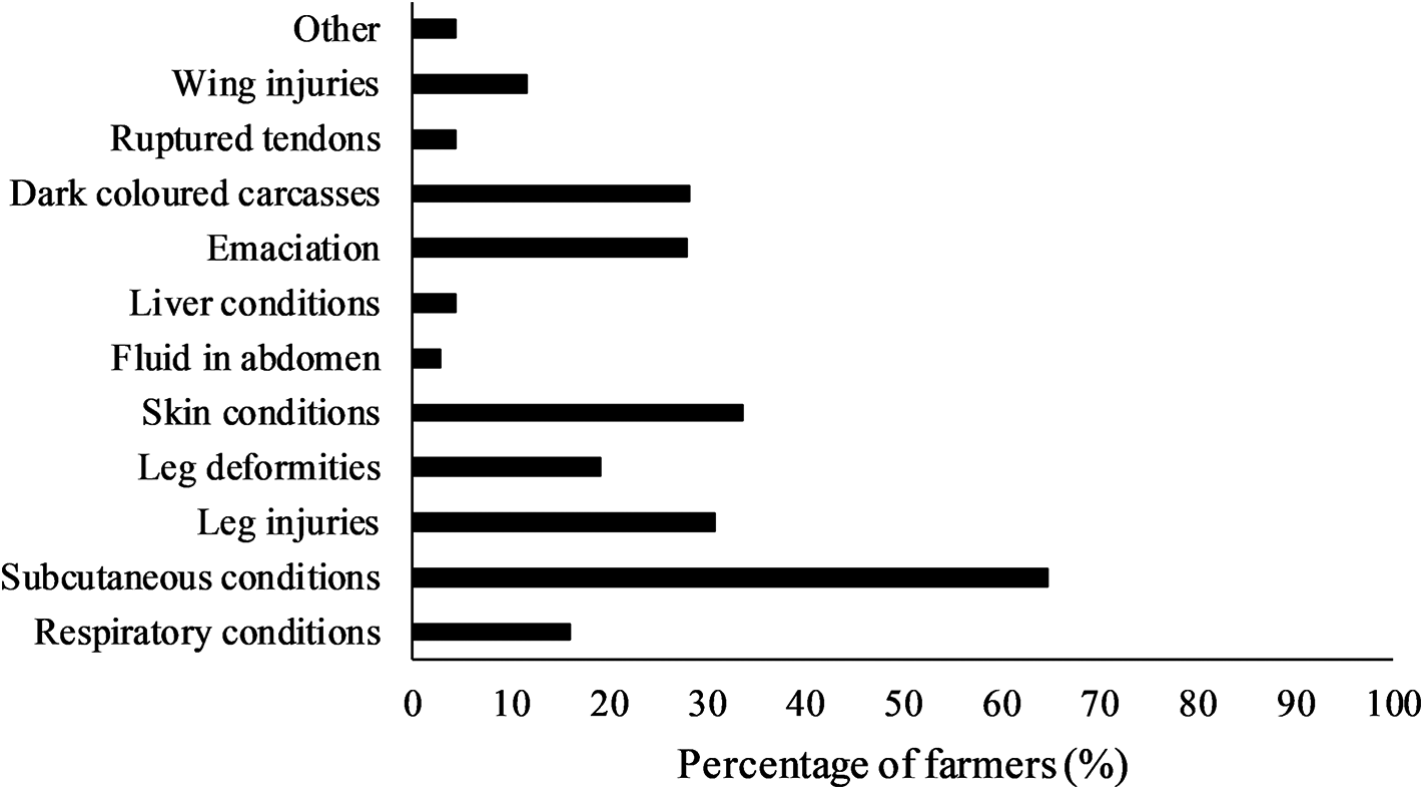
Percentage (%) of farmers (*n* = 68) that selected different causes of carcass condemnations and downgrading of turkeys during processing based on slaughterhouse records. Farmers could select multiple options.

Finally, farmers were also asked to select the most common reasons for carcass condemnations or downgrading of carcass value during processing as shown in Figure 3. The main reasons were related to skin (33.8%) or subcutaneous conditions (64.7%), as well as dark-colored carcasses (28.4%) and emaciation (27.9%). Leg injuries (30.9%) and leg/feet deformities (19.1%) were also mentioned frequently. Other injuries such as wing injuries (11.8%) and ruptured tendons (4.4%) were less common, and few mentioned transport issues or dead-on-arrivals (4.4%, “other”). Viscera-related issues such as respiratory conditions (16.2%), liver conditions (4.4%), and fluid in the abdomen (2.9%) were also reported (Figure 3).

### Perception of Turkey Farmers on Health and Welfare-Related Issues on Turkey Farms

Farmers were asked to rate the following issues from being no issue, a small issue, or a big issue on their farm during winter or summer: mortality, aggressive pecking, disease, leg injuries, leg deformities, breast injuries, and varying body size with results shown in Table 1. No apparent differences were observed between farmers' ratings between winter or summer for the majority of issues (1 within the 95% CI). Only aggressive pecking had greater odds of receiving a higher issue rating in summer compared to winter (OR > 1).

**Table 1:** Perceptions of farmers in ranking the importance of health and welfare-related problems as no issue, a small issue or a big issue on their farm during winter or summer.

	***n***	**Percentage of respondents**			**Statistics**	
		**No issue at all**	**A small issue**	**A big issue**	**OR**	**95% CI**
**Mortality**						
Winter	77	24.68	66.23	9.09	*Ref*.	*Ref*.
Summer	77	25.97	66.23	7.79	0.9	0.44 - 2.05
**Aggressive Pecking**						
Winter	78	43.59	52.56	3.85	*Ref*.	*Ref*.
Summer	78	35.90	47.44	16.67	2.8	1.28–6.00
**Disease**						
Winter	73	57.53	41.10	1.37	*Ref*.	*Ref*.
Summer	74	62.16	37.84	0.0	0.8	0.35–1.85
**Leg Injuries**						
Winter	73	36.99	57.53	5.48	*Ref*.	*Ref*.
Summer	76	39.47	55.26	5.26	0.9	0.40–1.90
**Leg Deformities**						
Winter	75	34.67	52.00	13.33	*Ref*.	*Ref*.
Summer	76	30.26	53.95	15.79	1.4	0.67–3.12
**Breast Injuries**						
Winter	72	63.89	30.56	5.56	*Ref*.	*Ref*.
Summer	74	70.27	22.97	6.76	0.7	0.32–1.67
**Varying Body Size**						
Winter	73	41.10	53.42	5.48	*Ref*.	*Ref*.
Summer	73	39.73	54.79	5.48	1.1	0.48–2.42

*Odds ratio (OR) and their 95% confidence interval (95%CI) for a higher rating (indicating the condition being a larger issue) were calculated using the winter rating as the reference (ref.) for the comparison. An OR > 1 indicates that farmers were more likely to give a higher rating in summer compared to winter, whereas an OR <1 indicates that farmers were less likely to give a higher rating in summer compared to winter. OR where the value 1 fell within the 95%CI indicate conditions that were rated equally for winter or summer*.

### Perception of Turkey Farmers on Health and Welfare-Related Issues Within the Turkey Sector

Similarly, farmers were asked to rate these issues as being of no issue, a small issue or a big issue for the turkey production sector as a whole (Table 2). In general, higher percentages of farmers were observed to rate the different conditions as a big issue when they were specifically asked about their importance for the sector. Specifically, disease, leg deformities, and breast injuries were more likely to be more of an issue (higher rating, OR > 1), while leg injuries tended to be more of an issue (1 at the 95% CI) for the sector rather than for individual farms. Farmers did not rate the importance of mortality, aggressive pecking, or varying body size differently (1 within the 95% CI) when asked about their farm or the sector as a whole.

**Table 2:** Perceptions of farmers in ranking the importance of health and welfare-related problems as no issue, a small issue or a big issue on their farm or for the sector.

	***n***	**Percentage of respondents**			**Statistics**	
		**No issue at all**	**A small issue**	**A big issue**	**OR**	**95% CI**
**Mortality**						
Farm	77	25.33	66.23	8.44	*Ref*.	*Ref*.
Sector	70	25.71	54.29	20.00	1.9	0.97–3.85
**Aggressive Pecking**						
Farm	78	39.75	50.00	10.26	*Ref*.	*Ref*.
Sector	69	37.68	47.83	14.49	1.3	0.65–2.60
**Disease**						
Farm	73	59.85	39.47	0.69	*Ref*.	*Ref*.
Sector	68	45.59	33.82	20.59	4.8	2.33–9.71
**Leg Injuries**						
Farm	73	38.23	56.40	5.37	*Ref*.	*Ref*.
Sector	66	31.82	53.03	15.15	2.1	1.06–4.35
**Leg Deformities**						
Farm	72	32.47	52.98	14.56	*Ref*.	*Ref*.
Sector	69	24.64	47.83	27.54	2.7	1.35–5.41
**Breast Injuries**						
Farm	72	67.08	26.77	6.16	*Ref*.	*Ref*.
Sector	67	43.28	35.82	20.9	4.9	2.42–9.78
**Varying Body Size**						
Farm	73	40.42	54.11	5.48	*Ref*.	*Ref*.
Sector	67	34.33	53.73	11.94	1.8	0.89–3.74

*Odds ratio (OR) and their 95% confidence interval (95%CI) for a higher rating (indicating the condition being a larger issue) were calculated using the farm rating as the reference (ref.) for the comparison. An OR > 1 indicates that farmers were more likely to give a higher rating for the sector compared to their farm, whereas an OR <1 indicates that farmers were less likely to give a higher rating for the sector compared to their farm. OR where the value 1 fell within the 95%CI indicate conditions that were rated equally for the farm or sector*.

## Discussion

Despite the importance of farmers and their role as primary caretakers of animals, little research has been conducted on what farmers feel are important turkey health and welfare issues on their farm, and their views have up until recently often been overlooked (29, 30). Farmers' perceptions of issues on their farm impact their health and well-being but also that of their animals, with further implications for food safety, farm productivity and sustainability—an interconnectedness highlighted by the OneWelfare concept (3). This survey presents the first insights into Canadian farmers' perceptions on health and welfare-related issues in turkey production. Understanding issues farmers face can help concentrate research efforts to overcome these challenges through, e.g., improved management practices or breeding.

It is important to keep in mind that the results described in this study reflect the number of farmers who indicated certain issues, and not the actual prevalence of issues among or within flocks. This complicates comparisons to other studies. Furthermore, different proportions of hen and tom flocks were represented through the farmers, and this likely influenced their perception of the different health and welfare conditions (31). In the present study, the most common reasons for culling included leg deformities and leg injuries, which, when taken together, were mentioned by nearly 90% of the farmers. Other frequently mentioned reasons for culling by over half of the farmers included sick birds (unrelated to leg issues), or birds with small body size relative for their age. These issues are similar to unthriftiness/disease and injuries mentioned as the main reasons for culling in an earlier survey among 57 turkey farmers in Canada (32). These issues are associated with reduced welfare in turkeys (11) and should be dealt with in a timely manner. Considering that farmers also frequently mentioned disease or ‘unknown’ as reasons for mortality on-farm, it is possible that not all birds are culled in a timely manner. The Canadian Code of Practice and Turkey Farmers of Canada Flock Care Program provide guidelines to ensure euthanasia is performed without delay and stresses that personnel should be trained to properly perform euthanasia (27, 33). However, research on decision-making around euthanasia indicates that this can be difficult for farmers and is dependent on euthanasia plans on-farm, clearly defined end-points, training, and farmer attitudes (34–36). Similarly, the Code of Practice acknowledges that euthanasia methods should consider the size and weight of turkeys, and the skill and comfort level of the person performing the procedure (27).

Leg problems in turkeys are consistently ranked as a top concern by veterinarians in the US, which includes lameness, footpad dermatitis, and leg deformities (37). Leg deformities, more so than leg injuries, were considered an important issue within the current study. Limb bone deformities, such as valgus or varus, are heritable (28). Their presence can be a side-effect of genetic selection for increased productivity. However, Hocking (28) states that this association is sometimes exaggerated, and advances have been made to reduce leg problems through genetic selection. The use of index selection to account for both productivity and leg health is suggested to improve both these aspects simultaneously (38, 39). Hocking (28) cautioned that it takes time for improvements in breeding flocks to translate to commercial flocks, and that independent assessments are needed to demonstrate that this change has occurred. Results from the current study suggest that for most farmers, it is still an issue that deserves attention. While much work has focused on genetic improvements, it should be acknowledged that farmers can feel that this is outside of their control (22). Other management strategies that appear most effective in controlling or reducing leg deformities in turkeys are changes in nutrition, lighting, flooring, and health management (40–42).

Culling for small body size can be linked to dehydration as small birds are unable to reach the feeders or drinkers (27). While this survey did not elucidate the reasons for this lack of flock uniformity or reasons why birds were unable to access drinkers, it highlights an important issue. It is also possible that dehydration occurs due to birds with leg issues being unable to reach the drinkers (11), showing the complex interactions between the potential factors or reasons for culling and mortality. Reasons for culling or mortality should be recorded as they can help identify relationships and management practices that can be improved (27).

Pecking injuries and cannibalism were other common reasons for culling (37.5%) and mortality (40.8%) mentioned by farmers. Duggan et al. (43) found that ~58% of all mortalities and culls observed in eight tom turkey flocks were due to pecking injuries. Assessment of turkeys at slaughter also revealed an average within-flock prevalence of feather pecking injuries of 6.6% and an average within-flock prevalence of head injuries of 0.1% (44). Furthermore, Allain et al. (44) also reported positive correlations between feather pecking injuries on the carcass and leg problems such as toe deviations and footpad swelling. Birds with leg injuries might be less able to escape and thus become the victim of injurious pecking (11).

Carcass condemnations and downgrading in turkey production are indicators of health and welfare issues and economic losses (12). The main reasons for condemnations or downgrading indicated by farmers in this survey included subcutaneous conditions, skin conditions, dark-colored carcasses, and emaciation. This is in line with the 2019 turkey condemnation report for federally inspected plants in Canada, where subcutaneous conditions, emaciation, dark-colored carcasses, and bruising were indeed the most common reasons for condemnations (45). Some of these conditions are likely associated with one another, as dark-colored carcasses (or cyanosis) was more common in flocks with emaciated birds (46). An older survey of a processing plant in Ontario also revealed that most trimming was due to bruising or fractures of wings and legs (47). Leg injuries and deformities were also frequently mentioned as reasons for carcass condemnations and downgrading in the current study and Canada overall in 2019 (45). Interestingly, the risk of condemnation at slaughter was higher in flocks which reportedly had leg disorders during rearing (16). Apart from a few studies (16, 46, 47), little is known about rates and reasons for condemnation or downgrading in turkey production, and their economic implications. The results from meat inspections can potentially provide information about the health and welfare of farm animals (48–50), and recent studies have suggested to include this in breeding programs (51, 52).

When farmers were asked to rate specific health- and welfare-related issues on their farm, mortality and leg deformities/injuries were considered an issue, followed by aggressive pecking and varying body size. In contrast, disease and breast injuries were not considered an issue on their farm by most respondents. With regard to disease, farmers may be presenting a favorable self-image, as can be the case with any self-reporting (53). Alternatively, farmers seem confident in their ability to control it through biosecurity, vaccination, and medication (54). However, the lack of approved efficacious drugs was highlighted as a risk for the turkey sector in the US (37), and in Canada as well antimicrobial use is being limited (55). In terms of breast injuries, it is likely that this is less visible on-farm, but later noticed at processing after defeathering (56), and is likely only of importance once carcasses are condemned for this reason (45). However, despite not being considered an issue on-farm, management practices such as, e.g., litter and floor management can contribute the occurrence of breast injuries which can cause pain and discomfort for birds (18, 56). Furthermore, skin conditions including breast buttons/blisters (inflamed bursa) were actually one of the more commonly mentioned reasons for condemnation or downgrading at slaughter in the current study. This highlights that breast injuries in turkeys should not be disregarded. Interestingly, the majority of the health and welfare-related conditions (with the exception of mortality, aggressive pecking, and varying body size) were considered more of an issue for the turkey production sector as a whole rather than individual farms. Aggressive pecking was considered a more significant issue in summer by some farmers. As the current survey was part of a larger project aimed at reducing injuries due to aggressive pecking it is possible that especially farmers with this issue participated. The higher importance of most issues for the sector can be linked again to social desirability (53) or farmers' understanding that these issues can influence the public perception of turkey farming.

It should be acknowledged that the responses of these 83 farmers should not be generalized to the entire sector, but instead represent ~20% of the turkey farmers in Canada. As the data were collected in a larger project with a different overall aim, namely to identify risk factors for footpad dermatitis and pecking injuries in turkey flocks, we have to acknowledge the possibility of responses being somewhat biased. Additionally, questions regarding the perception of turkey farmers were limited in the sense that through a self-administered (semi-)closed questionnaire one cannot collect as much detailed information on participants' beliefs and attitudes as in, for example, focus groups or face-to-face interviews (57). Therefore, the views expressed here should be considered with caution. However, the possible answers to the questions were comprehensive and determined in consultation with poultry welfare experts and poultry industry representatives. Furthermore, farmers had an option to write in their own response and comments. As such, we believe this work provides a good first insight into farmers' perceptions.

## Conclusion

This study provides the first insights into farmers' perceptions of health and welfare-related conditions in turkey production. Mortality, though attributed to different conditions, was considered an important issue for the sector by the majority of farmers. Specifically, leg deformities and leg injuries, varying body size, disease, and aggressive pecking were important issues which are likely interconnected. These results illustrate the need for research and an integrated approach between breeding companies, veterinarians, policy makers, and farmers to address these issues. This will ultimately benefit the health and well-being of both turkey farmers and turkeys.

## Data Availability Statement

The raw data supporting the conclusions of this article will be made available by the authors, without undue reservation.

## Ethics Statement

The studies involving human participants were reviewed and approved by University of Guelph Research Ethics Board. The patients/participants provided their written informed consent to participate in this study.

## Author Contributions

NS, EL, BW, AH-M, and CB conceived and designed the study. NS and EL conducted the study. NS analyzed the data and wrote the main manuscript. All authors reviewed and approved the final manuscript.

## Conflict of Interest

BW was an employee of Hybrid Turkeys at time of the study. The remaining authors declare that the research was conducted in the absence of any commercial or financial relationships that could be construed as a potential conflict of interest.
